# Bayesian Estimation of Pneumonia Etiology: Epidemiologic Considerations and Applications to the Pneumonia Etiology Research for Child Health Study

**DOI:** 10.1093/cid/cix144

**Published:** 2017-05-27

**Authors:** Maria Deloria Knoll, Wei Fu, Qiyuan Shi, Christine Prosperi, Zhenke Wu, Laura L. Hammitt, Daniel R. Feikin, Henry C. Baggett, Stephen R.C. Howie, J. Anthony G. Scott, David R. Murdoch, Shabir A. Madhi, Donald M. Thea, W. Abdullah Brooks, Karen L. Kotloff, Mengying Li, Daniel E. Park, Wenyi Lin, Orin S. Levine, Katherine L. O’Brien, Scott L. Zeger

**Affiliations:** 1 Department of International Health, International Vaccine Access Center,; 2 Department of Biostatistics,; 3 Department of International Health, and; 4 Department of Population, Family and Reproductive Health, Johns Hopkins Bloomberg School of Public Health,; 5 Department of Rheumatology, Johns Hopkins School of Medicine, and; 6 Division of Infectious Disease and Tropical Pediatrics, Department of Pediatrics, Center for Vaccine Development, Institute of Global Health, University of Maryland School of Medicine, Baltimore;; 7 Department of Biostatistics, University of Michigan, Ann Arbor;; 8 Kenya Medical Research Institute–Wellcome Trust Research Programme, Kilifi;; 9 Division of Viral Diseases, National Center for Immunizations and Respiratory Diseases, and; 10 Division of Global Health Protection, Center for Global Health, Centers for Disease Control and Prevention, Atlanta, Georgia;; 11 Global Disease Detection Center, Thailand Ministry of Public Health–US Centers for Disease Control and Prevention Collaboration, Nonthaburi;; 12 Medical Research Council Unit, Basse, The Gambia;; 13 Department of Paediatrics, University of Auckland,; 14 Centre for International Health, University of Otago, Dunedin,; 15 Department of Pathology, University of Otago, Christchurch, and; 16 Microbiology Unit, Canterbury Health Laboratories, Christchurch, New Zealand;; 17 Department of Infectious Disease Epidemiology, London School of Hygiene & Tropical Medicine, United Kingdom;; 18 Medical Research Council: Respiratory and Meningeal Pathogens Research Unit, and; 19 Department of Science and Technology/National Research Foundation: Vaccine Preventable Diseases Unit, University of the Witwatersrand, Johannesburg, South Africa;; 20 Center for Global Health and Development, Boston University School of Public Health, Massachusetts;; 21 International Centre for Diarrhoeal Disease Research, Bangladesh, Dhaka and Matlab;; 22 Milken Institute School of Public Health, Department of Epidemiology and Biostatistics, George Washington University, Washington, DC;; 23 Bill & Melinda Gates Foundation, Seattle, Washington

**Keywords:** Bayes theorem, etiologic estimations, pneumonia, epidemiologic methods, statistical models.

## Abstract

In pneumonia, specimens are rarely obtained directly from the infection site, the lung, so the pathogen causing infection is determined indirectly from multiple tests on peripheral clinical specimens, which may have imperfect and uncertain sensitivity and specificity, so inference about the cause is complex. Analytic approaches have included expert review of case-only results, case–control logistic regression, latent class analysis, and attributable fraction, but each has serious limitations and none naturally integrate multiple test results. The Pneumonia Etiology Research for Child Health (PERCH) study required an analytic solution appropriate for a case–control design that could incorporate evidence from multiple specimens from cases and controls and that accounted for measurement error. We describe a Bayesian integrated approach we developed that combined and extended elements of attributable fraction and latent class analyses to meet some of these challenges and illustrate the advantage it confers regarding the challenges identified for other methods.

Studies aimed at determining the cause of pneumonia have inherent analytic challenges because they need to incorporate multiple types of laboratory measurements that have imperfect sensitivity and specificity for identifying the causative agent. This is because such studies may evaluate multiple specimens with multiple assays to improve sensitivity for each pathogen being tested, may include control children who do not have pneumonia to provide information on the test specificities, and may test for many pathogens to reveal potential causes for a greater number of cases. However, there are no existing analytic methods that can integrate such diverse data and account for their imperfect sensitivity and specificity to estimate the proportion of cases caused by each pathogen (ie, the etiologic distribution or etiology pie). Previous analytic approaches, which include expert review and latent class analysis for case-only data and logistic regression and attributable fraction for case–control studies, have ≥1 serious limitations (Figure 1), described in detail by Hammitt et al [1]. In addition, all of these methods intrinsically assume 100% sensitivity of the tests; although the expert review and attributable fraction approaches could account for sensitivity post hoc, there is no principled way to incorporate uncertainty around the sensitivity estimates.

**Figure 1.**
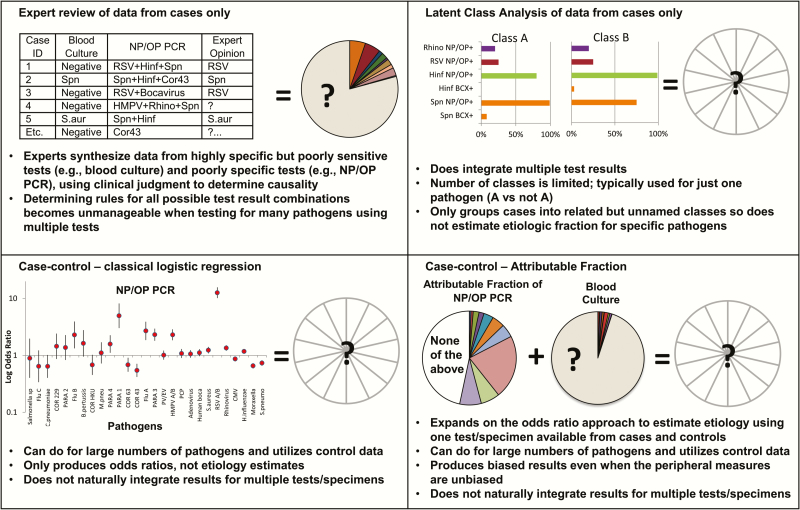
Alternative analytic approaches used for determining pneumonia etiology. Abbreviations: BCX+, positive blood culture; Cor, coronavirus; Hinf, *Haemophilus influenzae*; HMPV, human metapneumovirus A/B; NP/OP, nasopharyngeal/oropharyngeal; PCR, polymerase chain reaction; Rhino, rhinovirus; RSV, respiratory syncytial virus; S. aur, *Staphylococcus aureus*; Spn, *Streptococcus pneumoniae*.

Previous pneumonia studies that conducted multiple tests on cases and controls have acknowledged the limitations of the available analytic choices when attempting to integrate these data [2, 3]. Because of insufficient epidemiologic and statistical tools, those investigators did not attempt to estimate the pneumonia etiologic distribution. Instead, they described etiology for a subset of the cases where they were confident about the etiology, such as blood or lung aspirate culture-positive cases, or for a subset of pathogens where measurements were considered both sufficiently sensitive and specific, such as for tuberculosis and pertussis, then designated all remaining cases to have “unknown” etiology [1]. Thus, pneumonia etiology studies need an approach that can better integrate data from multiple sources.

The Pneumonia Etiology Research for Child Health (PERCH) study is a case–control study to estimate the etiology of severe and very severe hospitalized pneumonia among children in 7 lower- and middle-income African and Asian countries [4]. PERCH aimed to collect as much data to inform etiology as possible using state-of-the-art diagnostic testing methods and standardizing clinical and laboratory procedures across all sites. Nasopharyngeal/oropharyngeal (NP/OP) specimens were tested by polymerase chain reaction (PCR) for >30 pathogens in both cases and controls; whole blood was tested for pneumococcus by PCR in both cases and controls; blood cultures were performed in cases, detecting many types of bacterial pathogens; induced sputum collected from cases was tested for tuberculosis by culture; and select cases had lung aspirates or pleural fluid collected and tested by culture and PCR [5, 6]. Each of these measurements has the potential for error, either in sensitivity or specificity.

In this article, we describe a novel analytic approach used by the PERCH study, referred to as the PERCH integrated analysis, designed to address the challenges of incorporating this multidimensional array of evidence to estimate the etiologic distribution of pneumonia cases. We compare the PERCH integrated analysis to attributable fraction, the current main alternative method, under conditions relevant for attributable fraction and then illustrate the performance of the PERCH integrated analysis under conditions expanded beyond the limits of attributable fraction.

## OVERVIEW OF THE PERCH INTEGRATED ANALYSIS

We begin by contrasting the PERCH integrated analysis to attributable fraction, a common approach for analyzing case–control data. Although there are other approaches to analyze etiology data as described, attributable fraction is the only one capable of reproducibly estimating an etiologic distribution using case–control data (albeit only with 1 specimen).

### Attributable Fraction Approach

The attributable fraction method for assessing pneumonia etiology is described elsewhere [1], but briefly it compares case and control results from a single specimen using the odds ratio (OR) and determines the proportion of all pneumonia cases attributable to a pathogen by multiplying the attributable fraction = 1−(1/OR) by the proportion of cases positive for that pathogen (ie, estimates etiology for the population of cases, but not for each individual case) [1, 7]. When multiple etiologies are being estimated, simulation experiments (pathogens A and B in Supplementary Figure 1) show that the coefficients are biased as a result of measurement error and inherent negative correlation among the predictors because in this simulation only 1 predictor can be positive (ie, in a case where pathogen A is the true cause, pathogen A will more likely be positive and the other pathogens will more likely be negative relative to other cases). Because pathogens with an odds ratio ≤1 result in a zero or negative attributable fraction, these pathogens do not appear in the etiologic distribution and are interpreted as not being causes of the disease. Attributable fraction analyses also typically do not account for imperfect sensitivity of the tests so implicitly assume a sensitivity of 100%; if the sum of attributable fractions across the multiple etiologies assessed is <100%, this implies that the remainder is due to other pathogens not tested for.

The PERCH integrated analysis has several appealing features for the PERCH study and other similar etiology studies that are not addressed by existing analytic choices [7, 8].

1. Incorporates multiple sources of specimen measurements such as culture and polymerase chain reaction of blood, nasopharyngeal swabs, lung aspirates, and pleural fluid.
2. Estimates the etiologic fractions for each individual case in addition to the population etiology pie, which means that among all the cases testing positive for a pathogen, the PERCH integrated analysis determines the probability for that individual’s data profile of each pathogen being attributed as the cause of the pneumonia.
3. Does not require a pathogen’s odds ratio to be >1 for that pathogen’s data to be included in the analysis.
4. Enables incorporation of useful existing (prior) clinical knowledge regarding the sensitivity (true positive rate) of each measurement.
5. Uses the new evidence provided by the data about sensitivity (true positive rate) of each measurement, and combines it with the prior knowledge to produce revised sensitivity estimates (with confidence intervals) that reflect all the evidence.
6. Incorporates the uncertainty about the sensitivities and specificities into the uncertainties for the estimated etiologic fractions.
7. Imputes missing data by treating the missing values as unobserved, which means that cases and controls with partial data are not excluded from analyses but instead can contribute the evidence they do have.
8. Allows for combining pathogen-specific etiology results into total viral and/or bacterial etiology fractions along with their credible intervals.

### The PERCH Integrated Analysis Approach

**Figure 2.**
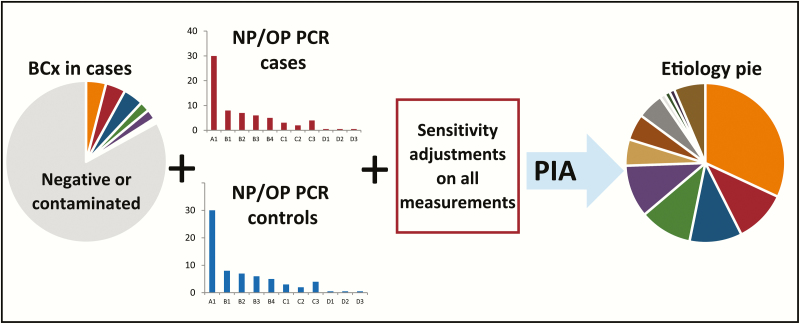
Estimating the etiologic fraction from a study with 2 types of measurements, 1 with control data, and accounting for imperfect sensitivity of both measurements: the PERCH integrated analysis method. The PERCH integrated analysis can combine multiple specimens (shown here for 2 but can integrate more specimen/test measurements, such as whole-blood polymerase chain reaction [PCR] and lung aspirate culture and PCR) and adjust each measurement for pathogen-specific sensitivity to estimate the etiologic fraction using all available evidence. Abbreviations: BCx, blood culture; NP/OP PCR, nasopharyngeal/oropharyngeal polymerase chain reaction; PIA, PERCH integrated analysis.

The PERCH integrated analysis addresses the 3 major limitations of the attributable fraction approach: it allows incorporation of results from multiple tests, incorporates adjustment for imperfect sensitivity of each test, and estimates etiology for individual cases (**Text Box** and Figure 2) [8, 9]. The analysis uses Bayesian methods [10] that allow incorporation of current knowledge of sensitivity, including estimates of its uncertainty. Prior knowledge about sensitivities is required to estimate the etiology when there are no gold-standard tests. As such, the assumptions regarding the sensitivity estimates of each test are identified, quantified, and open to scrutiny rather than overlooked. Like latent class analysis, the PERCH integrated analysis treats each child’s true cause as a latent variable and then assumes each test provides evidence about that cause. In this way, it enables integration of information from multiple data sources, including case-only data and case−control data, to estimate probabilities at the individual case level.

#### Integrating Multiple Measurements, Sensitivity, and Specificity

The concept of how the PERCH integrated analysis integrates multiple measurements while simultaneously accounting for the distribution for the unknown sensitivity can be illustrated by considering data from 2 tests for 1 pathogen. Each test alone provides an estimate of the etiologic fraction of the pathogen. Integration can be performed by averaging the results to obtain a single overall estimate. One could conceive that averaging the results in a way that draws on the strengths of each may be an acceptable method to get a single overall estimate that incorporates the evidence from each test.

We illustrate using an example common to pneumonia etiology studies of integrating NP/OP PCR results from cases and controls with blood culture data from cases to estimate the etiologic fraction due to pneumococcus. Let us assume we observed 9 of 600 (1.5%) hospitalized cases with pneumococcus detected by blood culture, and based upon prior data from vaccine probe trials, we assume that blood culture sensitivity is between 5% and 20%, with 95% probability. After accounting for the false negatives, we would estimate the proportion of cases with pneumococcal pneumonia to be between 7.5% and 30% (ie, 7.5% of cases if the sensitivity is 20%, and 30% of cases if the sensitivity is 5%). For NP/OP PCR data, let us assume that high density NP/OP colonization with pneumococcus is a proxy for pneumococcal pneumonia but with imperfect specificity that can be estimated using the control data. Suppose that in the cases, we detected 30% with high-density NP/OP pneumococcal colonization and observed odds ratio of 2 when compared with controls. Using the attributable fraction method, we would estimate 15% of cases to have pneumococcal pneumonia if we assume NP/OP PCR test sensitivity is 100% (ie, 1−[1/OR = 2] × 30%). However, the study data provide evidence regarding sensitivity of NP/OP PCR, which is the proportion of the 9 blood culture–positive pneumococcal cases that also had high-density pneumococcal colonization. As with the first test, we also use prior knowledge about the sensitivity of the NP/OP PCR. Suppose that the observed rate of concordance of the 2 tests, combined with the prior knowledge, leads us to conclude that the true NP/OP PCR sensitivity is in the range 50%–90% with high probability. If so, then between 17% and 30% (ie, 15%/90% and 15%/50%) of cases would be estimated to have pneumococcal pneumonia based on the NP/OP PCR data. Note that although the estimates from the 2 measurements are not identical, they are relatively consistent in that both support estimates in the range of 17%–30% (ie, the overlapping range from the 2 data sources).

The PERCH integrated analysis provides a method that integrates these 2 estimates, the 1 with greater specificity but wide uncertainty due to small sample size (blood culture) and the 1 with the larger (more statistically stable) number of positive results but with poor specificity (NP/OP PCR). The PERCH integrated analysis also incorporates statistical uncertainty of the observations (eg, if we repeated the study we might have observed 7 or 11 positive blood cultures instead of 9) and the statistical uncertainty of sensitivity and does this for all pathogens at once.

#### The Bayesian Concept

Bayesian analyses are similar conceptually to how a clinician approaches a pneumonia patient regarding the etiology of their condition. They do so with an informed set of prior expectations based on the season of the year, knowledge about common causes of pneumonia, the age of the patient, and so forth. Likewise, in a Bayesian analysis, the study team must provide a set of principled evidence-based expectations about the state of the infection in the population of the cases (ie, etiology and sensitivity “priors”), before incorporating the evidence from the observed data. Stating the assumptions regarding the sensitivity of each measure as was done above is an example of setting sensitivity priors. If there were no prior information to suggest that 1 pathogen is more likely responsible for the pneumonia over another for any given case, then setting every pathogen as equally likely is an example of an etiology prior. More technical details on the PERCH integrated analysis methodology are presented in the PERCH Integrated Analysis Statistical Methods section and in Table 1, and this is followed by simulation analyses demonstrating its advantage over attributable fraction.

**Table 1.** Description of the Key Parameters Requiring Specification Used in the PERCH Integrated Analysis Approach

Input parameter	**PIA application**	**Implications for selecting values for PIA parameters**	**Application in other settings**
**Pathogens**	All pathogens included in the analysis will be included in the final etiologic distribution estimate; although some estimates may get near 0%, none is ruled out as definitely not a causative pathogen.	Determined on the basis of biological relevance to the disease, implying included pathogens are believed to be causative organisms. It is important not to include noncausative pathogens; although any individual pathogen’s estimate may be close to 0, the cumulative etiologic fraction across many noncausative pathogens may be nontrivial. Because all etiologic fractions must sum to 100%, their inclusion will result in underestimation of the remaining true causative pathogens.	Attributable fraction: Organisms with measurements lacking association with case status are assigned 0% etiologic fraction (ie, ruled as definitely not a causative pathogen), even though the lack of association may be due to small sample size or true pathogens that are commonly observed in controls.
**Etiology fraction priors**	Knowledge about the likely infection status of the cases before any data are available (prior probabilities or etiology fraction priors). Evidence in the observed data shifts the etiologic fractions away from the prior values to the final estimates.	Determined by 3 characteristics: informativeness, uncertainty, and distribution. If the goal is to summarize the current study evidence with as little influence from external evidence or opinion as possible, then all pathogens would be considered equally likely, the uncertainty would be wide, and the distribution would be flat.	Clinical: Similar to how a clinician approaches a pneumonia patient with a set of prior beliefs regarding their etiology.
**Pathogen-informative vs -noninformative**	Pathogen-noninformative etiologic priors assign each organism the same etiologic fraction distribution at the outset. Pathogen-informative priors assign etiologic fractions based on evidence from data external to the current study.	Choice depends on how results are to be interpreted: Noninformative: Results predominantly reflect the current study data alone. All pathogens would be considered equally likely to be causative—that is, the etiology prior = 100% divided by the number of pathogens (ie, if 10 pathogens, then each is assigned a prior etiologic fraction of 10%). Informative: Results reflect all available information, both study data and external evidence (similar to a meta-analysis). The choice may be made at the organism level, enabling use of data from past studies even when only available for a subset of pathogens.	Clinical: Knowledge of seasonality of pathogens or etiology of previous patients may sway beliefs to be more in favor of some pathogens over others (informative) rather than equipoise (noninformative).
**Uncertainty**	Refers to the probability distribution around the etiologic prior value for each pathogen.	In general, the impact of the etiology fraction prior for a given pathogen is related to both the level of uncertainty and the strength of evidence in the data, whether for that organism or against that organism due to strong evidence for other organisms. When the probability distribution is wide (ie, uncertain), less evidence in the data is required to shift the etiologic fraction from the prior value.	Clinical: A decision to treat without testing or to test in spite of an ongoing epidemic of a particular pathogen are expressions of uncertainty in the clinical setting.
**Distribution**	A Dirichelet prior is used to describe the distribution of the etiologic fractions of the pathogens (Supplementary Figure 2) [9]. It influences (1) the likelihood of each pathogen to be the cause for a given child and (2) the distribution of the etiologic fractions across the population of cases (ie, whether a few pathogens are responsible for most disease vs all pathogens cause an equal fraction of disease).	A minimally informative Dirichelet prior is one where each pathogen has an equally likely chance of being the cause for a given child.	Clinical: During an epidemic, a physician may assume that a case is more likely caused by the epidemic pathogen than any other pathogen.
**Sensitivity priors** (Sensitivity: Probability of detecting a pathogen in a specimen [eg, blood] given that pathogen is in the site of infection [eg, the lung for pneumonia])	Sensitivity priors are set separately for each silver- and bronze-standard measurement for each specimen/test/pathogen combination.	Because the primary analysis goal is to determine etiology, it is important to use as much information about sensitivity as is available (ie, informative priors) because the model estimates both etiology and sensitivity simultaneously and cannot estimate etiology without a sensitivity distribution. Note: Specificity (ie, 1 – probability of detecting the pathogen in the specimen given that pathogen is not in the infection site of interest) is estimated using data from the controls, not with priors.	All analytic methods require assumptions about the sensitivity of the measurements to estimate etiologic fraction [1]. Attributable fraction: Assumes that test results are 100% sensitive, an assumption known to be in error for most measurements, but the resulting bias is not quantifiable. Expert review of cases: Typically acknowledges imperfect sensitivity but does not account for it quantitatively. Both methods can account for test sensitivity by dividing the results by the test sensitivity, but they are unable to assign the adjusted fraction to individual cases or to account for uncertainty.
Uncertainty range	Each sensitivity prior is specified as a range of plausible values rather than as a single point estimate. The range represents 95% of the prior’s distribution (ie, not min-max) and allows posterior estimates outside of this range to occur.	A plausibility range of values is selected to reflect our inherent uncertainty about the exact value of sensitivity. Wider ranges in the measurement’s sensitivity prior result in more uncertainty in the etiology estimate.	Other analytic methods that have accounted for sensitivity use only a point estimate and therefore do not incorporate its uncertainty.
Informative vs noninformative	Noninformative priors are those at the full range of 0–100% that give equal probability to every sensitivity value (ie, uniform distribution). Informative priors narrow the range and draw on organism-/test-specific evidence from data external to the study.	Noninformative sensitivity priors introduce considerable uncertainty in the analysis, which, in turn, produces wide confidence intervals around the etiology fraction estimates. Where there is a general consensus, making even modest assumptions about the sensitivity of the measurements can improve the ability to estimate etiology. Sensitivity priors at the individual level may also be set to account for case-specific influential factors, such as antibiotic preexposure and specimen volume.	All other methods are informative because they select a single value, such as 100% sensitivity.
Distribution	A beta distribution is used with parameters that produce a unimodal distribution with more of its probability in the center of the uncertainty range (ie, as opposed to a uniform distribution).		Other analytic methods that have accounted for sensitivity use only a point estimate and therefore do not incorporate its probability across a range of values.

## PERCH INTEGRATED ANALYSIS STATISTICAL METHODS

Technically, the PERCH integrated analysis is what Wu et al have called a nested partial latent class model, which acknowledges a case’s or control’s observations as error-prone measurements of their unobserved lung infection status [8, 9]. The “latent class” is the unknown etiology of the cases, and it is “partially” latent in a case–control analysis because the control’s lung status is known (no infection). The detection of a pathogen in a control specimen indicates a false-positive result for case etiology attribution, therefore providing direct specificity estimates for that test result. A nested partial latent class model assumes that the pathogen infecting a case’s lung is present and detected in each specimen with a given sensitivity of the test measurement. *Nesting* refers to extra latent subclasses within an etiologic class to account for situations where results of some measurements are not independent due to various biological or laboratory factors, such as poor specimen collection resulting in negative results for all pathogens measured. To estimate the etiologic fraction for each organism, the PERCH integrated analysis incorporates the etiology and sensitivity priors with the likelihood of the observed data through a numerical integration process (Markov chain Monte Carlo [MCMC] [11]) to obtain probability distributions of the etiology that comprise the main output from the model (ie, “posterior” distributions). The posterior is the prior distribution updated by the evidence measured by the study.

### Input Parameters Required for PERCH Integrated Analysis

Because the PERCH integrated analysis is a Bayesian analysis, it requires the user to specify 3 key parameters: (1) the pathogens that will be included in the etiology pie, (2) starting values for the distribution of the etiologic fractions (etiology prior distributions) that give pathogens equal or unequal weight (see below), and (3) the assumptions regarding the sensitivity (sensitivity prior distributions) for each of the multiple tests. As a Bayesian method, the key unknowns (etiologic fractions, sensitivities) are assumed to have probability distributions that represent our uncertainty about their values. The analysis starts with the user-specified prior distributions that reflect our degree of uncertainty before the study and are updated by the evidence in the data to produce posterior distributions reflecting our reduced uncertainty after the study. This approach uses the prior as a starting place. Although providing starting values for the distribution of the etiology fraction may seem counterintuitive because this is what we aim to learn from the study, in PERCH we choose “noninformative” priors that represent our starting assumption that each pathogen has an equal chance to be the cause for a particular child. With respect to prior assumptions, non-Bayesian methods also make them—for example, in the form of the analysis selected. A complete description is shown in Table 1.

The selected values for the priors need to be evaluated in the context of a study’s case definitions and eligibility criteria and whether they should differ across subgroups. The priors are distributions of plausible (allowable) values, not single values. For example, when setting the sensitivity prior to support an assumption of 50% sensitivity, the prior could be set with a range of 25%–75% to allow for error if the exact magnitude is imprecise. When there is truly no prior knowledge about sensitivity, its probability could be uniform across the range 0%–100%.

### Measurements

Gold-standard measurements are assumed to have both perfect sensitivity and specificity. Gold-standard measurements are rare in most situations, but perhaps PCR of pleural fluid from cases with pleural effusion is an example in pneumonia studies. Silver-standard measurements are assumed to have perfect specificity but imperfect sensitivity (eg, blood culture), and bronze-standard measurements have both imperfect sensitivity and specificity (eg, NP/OP PCR). Gold- and silver-standard measurements are only needed from cases because their specificity assumption would imply no control would be positive, whereas bronze-standard measurements are most useful when also available from controls to provide stronger evidence of specificity.

Organisms tested for may vary across measurement types. For example, in the PERCH study, some bacteria have both silver- and bronze-standard measurements, whereas other bacteria have silver-standard measurements only and viruses have bronze-standard measurements only. Organisms may also have multiple measurements of the same type; for example, cases and controls may have pneumococcal results from NP/OP PCR, NP culture, and blood PCR (ie, 3 bronze-standard measurements).

For simplicity, each measurement is incorporated into the analysis as a binary variable (positive or negative). Continuous measurements such as pathogen density are dichotomized using thresholds. Missing data are treated as unobserved parameters and are handled during the model estimation using standard Bayesian methods [10]. The same approach can be used with a mixture of continuous and binary measures at the expense of some additional complexity.

### Output

There are 2 main etiology outputs from the PERCH integrated analysis: an etiology distribution for the population of all cases and one for each individual case. The estimated population etiologic fraction for a given organism is approximately the mean of the individual case probabilities for that organism and has a distribution calculated using random samples from MCMC, from which a 95% credible interval (95% CI), the Bayesian analogue of the confidence interval, is calculated [10].

At the individual case level, the etiologic probability for each organism ranges from 0% to 100%; their sum across all the organisms will by definition be 100%. Individuals with an organism identified by a silver-standard measurement, which has 100% specificity, will have an etiology probability of 100% for that organism, with all remaining organisms estimated at 0%. For individuals with organisms identified by bronze-standard measurements only, their etiology probabilities will be estimated by incorporating the prevalence and strength of evidence at the population level in addition to the observed measurements for that individual (ie, cases positive only for that pathogen will have a higher probability of being attributed to that pathogen than cases positive for multiple pathogens).

The PERCH integrated analysis also provides as an output updated sensitivity estimates for the bronze- and silver-standard measurements. The data provide evidence of bronze-standard (eg, NP/OP PCR) sensitivity, which is then incorporated with the sensitivity priors. The evidence in the data comes from the proportion of cases with positive silver-standard (eg, blood culture) results (and gold-standard results, if available) for a given pathogen that are also bronze-standard–positive for that pathogen. However, unless there are gold-standard measurements, the data cannot directly inform sensitivity for silver-standard measurements for any pathogen or for bronze-standard measurements for organisms without silver-standard evidence. Nevertheless, the sensitivity estimates output for these measurements do get updated indirectly because the etiologic fraction estimate, case prevalence, sensitivity, and specificity must all align given the data across all pathogens. The posterior distribution of sensitivity is also summarized by a mean and 95% credible interval.

#### Bubble Plot

The output from a single PERCH integrated analysis is displayed in the form of a bubble plot. The bubble plot improves upon the box plot, the usual method to visualize estimates and their uncertainty, by drawing attention to pathogens with the most relative precision instead of those with the most uncertainty. The bubble plot does this by plotting a larger bubble for pathogens with less uncertainty (ie, the area of the bubble is proportional to the estimated etiologic fraction divided by its standard error), whereas the box plot presents the largest boxes for pathogens with the most uncertainty.

## SIMULATION ANALYSES

### Simulation Analyses of Etiology for a Single Specimen: PERCH Integrated Analysis Versus Attributable Fraction

We estimated etiology using both the attributable fraction and PERCH integrated analysis methods for conditions within the limits of attributable fraction methodology by analyzing results from just 1 specimen tested in both cases and controls. To quantify the magnitude and direction of any bias, we performed the analyses on simulated case and control data that reflected sensitivity and specificity of the tests and known etiology for the cases.

#### Methods to Simulate Data

**Figure 3.**
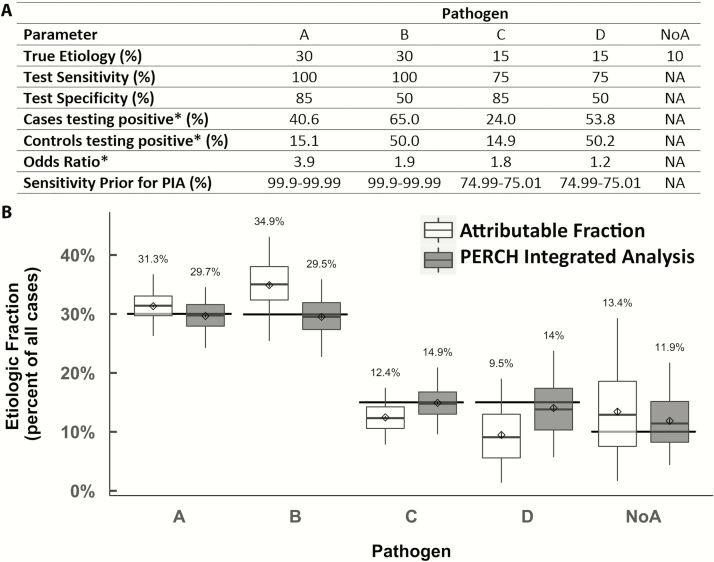
Analysis of 500 simulated datasets for a study that had only 1 specimen (A) and resulting etiologic fraction estimates using attributable fraction and PERCH integrated analysis methods (B). *A*, Analyses performed on measurements from 600 cases and 600 controls for each of 500 simulated datasets. *Prevalence and odds ratios estimated by averaging across the 500 datasets that were created based on the true etiology, sensitivity, and specificity values. *B*, Description of boxplots: Bold black line, mean of the true value across the 500 datasets; Diamond, average etiologic estimate across the 500 datasets; Vertical line through diamond, confidence interval around the average etiologic estimate; Boxplot, distribution of the etiologic estimates across the 500 datasets. Numbers above boxplots indicate numeric value of the diamond. Abbreviations: NA, not applicable; NoA, none-of-the-above; PIA, PERCH integrated analysis.

We created a simulated population where the “true” etiology of the cases was known for a simple scenario in which only 4 pathogens (A, B, C, and D) plus a “None of the Above Pathogens” category (NoA) are responsible for pneumonia. The true fractions of disease caused by each pathogen were 30%, 30%, 15%, and 15%, respectively, and 10% for NoA. We simulated test results for 600 cases and 600 controls given the known etiology for each case and used random sampling to apply the sensitivity and specificity values for each pathogen A–D (Figure 3A). We created varying combinations of sensitivity and specificity for each pathogen (test sensitivity was 100% for pathogens A and B and 75% for pathogens C and D; test specificity was 85% for pathogens A and C and 50% for B and D). We created 500 simulated random datasets and performed attributable fraction and PERCH integrated analysis on each, as if a clinical study was conducted 500 times.

#### Results of Attributable Fraction

The attributable fraction analysis performed made no adjustments for sensitivity (thus implicitly assuming 100% sensitivity). The mean (with 95% confidence interval) and selected quantiles from the distribution of the 500 etiologic fraction attributable fraction results for all 5 pathogen groups are shown in the white box plots of Figure 3B and in **Supplementary Table 1**. Under conditions when true sensitivity and specificity are both high (eg, 100% and 85%, respectively, for pathogen A), attributable fraction approximates the true pathogen prevalence but is biased because the sensitivity for some pathogens is misspecified (ie, assumes 100%). This inherent bias with attributable fraction is caused by the negative correlation between test results for different pathogens that are competing with one another to predict case status [7]. If odds ratios are estimated without adjusting for the results of the other pathogens, the resulting attributable fraction estimates are unbiased when sensitivity is 100% (blue box plots in **Supplementary Figure 1**). However, estimating single causes in the absence of data on other causes does not reflect the situation in pneumonia etiology studies.

Regardless of whether we are estimating etiology for a single or multiple pathogens, when sensitivity is <100% (pathogens C and D), the attributable fraction method underestimates etiology, and the magnitude of both the bias and variance increases as specificity decreases (ie, as the odds ratio approaches 1.0 as for pathogen D; white boxes in Figure 3B). The etiologic fractions of the pathogens are not constrained to sum to 100%, so the distribution will incorrectly sum to <100% when ≥1 pathogens have <100% sensitivity. Often the resulting underestimated etiologies will be misattributed to pathogens not tested for (NoA white boxes in Figure 3B), thus overestimating their true fraction. To account for sensitivity, the etiologic fractions could be expanded either by normalizing so that all pathogen-specific slices sum to 100%, which assumes the same sensitivity across all pathogens, or by dividing individual pathogens by their sensitivity. However, such adjustments are typically not done and there is not a natural, principled way to incorporate the uncertainty around the sensitivity estimates.

#### Results of the PERCH Integrated Analysis

The sensitivity priors for the PERCH integrated analysis were first set to be consistent with the truth to demonstrate how the PERCH integrated analysis performs under this ideal, but unrealistic, condition and then compared with more realistic, wider sensitivity prior ranges. Because the PERCH integrated analysis adjusted for imperfect sensitivity, the PERCH integrated analysis results were less biased than for attributable fraction for all combinations of sensitivity and specificity (ie, for all pathogens A–D; gray boxes in Figure 3B**; Supplementary Table 1**). As a result, the percentage attributed to NoA was also less biased when compared with attributable fraction and more frequently produced estimates that were closer to the true etiologic fraction (ie, narrower box plot). When the PERCH integrated analysis was performed using more realistic priors to reflect greater uncertainty in the sensitivity of the measurements (ie, wider ranges), the results were similar (**Supplementary Figure 3**). Sensitivity priors with wider ranges are also illustrated in the next simulation analysis involving multiple specimens.

### Simulation Analyses Using the PERCH Integrated Analysis of Etiology for Studies With Multiple Specimens

An advantage of the PERCH integrated analysis is that it naturally handles multiple measures for some or all of the pathogens. In this example, NP/OP PCR tested in both cases and controls and blood culture performed for cases only were analyzed. Again we quantified the magnitude and direction of bias by performing the analyses on simulated case and control data that reflected sensitivity and specificity of the tests and known etiology for the cases.

#### Methods to Simulate Data

**Figure 4.**
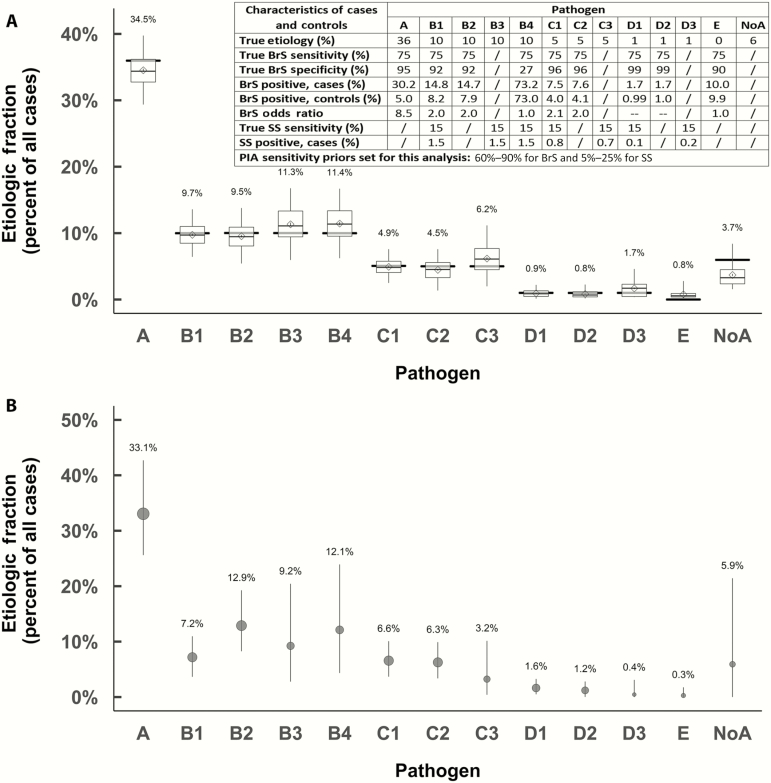
Etiologic fraction estimates using PERCH integrated analysis: distribution of results from analysis of 500 simulated datasets containing cases with known etiology (*A*) and results from 1 randomly selected dataset (*B*). *A,* Pathogens A through D represent true pneumonia-causing pathogens that were tested for, pathogen E represents a pathogen that was tested for but does not cause pneumonia, and NoA represents pathogens that cause pneumonia but were not tested for. Slashes in table indicate not applicable for the pathogen. Description of boxplots: Bold black line, mean of the true value across the 500 datasets; Boxplots display the distribution of etiologic fraction point estimates from 500 simulated datasets: Diamond, average etiologic estimate across the 500 datasets; Vertical line through diamond, confidence interval around the average etiologic estimate; Numbers above boxplots indicate the numeric value of the diamond; whiskers denote the 5th and 95th percentiles of the etiologic fraction point estimates. *B,* Bubble plot presenting the PERCH integrated analysis results from 1 randomly selected dataset. The area of the bubble is proportional to the estimated etiologic fraction (number above the bubbles) divided by its standard error (ie, the larger the bubble, the greater the degree of confidence in the estimate). Abbreviations: BrS, bronze-standard data (imperfect sensitivity and imperfect specificity; eg, nasopharyngeal polymerase chain reaction); NoA, none-of-the-above pathogens; PIA, PERCH integrated analysis; SS, silver-standard data (imperfect sensitivity and perfect specificity; eg, blood culture).

The true etiology fractions were specified to mimic a plausible scenario for a pneumonia etiology study in which 12 pathogens plus an NoA category (ie, pathogens not tested for) are responsible for pneumonia (Figure 4B). The NP/OP PCR data had imperfect sensitivity and specificity (ie, bronze standard), and blood culture data had perfect specificity but imperfect sensitivity (ie, silver standard). Pathogens varied by the type of data available in that some had both bronze- and silver-standard data (eg, bacteria); some had bronze-standard data only (eg, viruses); some had silver-standard data only (eg, bacterial pathogens found on blood culture that were not tested for in the NP/OP); and 1 had both bronze- and silver-standard data, but the bronze-standard data was uninformative (eg, bacterial pathogen with NP/OP PCR OR = 1.0 because it was commonly carried in all children). True NP/OP PCR sensitivity was 75% for all pathogens, but specificity varied by pathogen. True blood culture sensitivity was 15% for all pathogens. Simulated datasets containing 600 cases and 600 controls were created by random sampling from “populations” with the case and control pathogen prevalences produced based on the true etiology proportions and sensitivity and specificity values. We created 500 simulated datasets, and the PERCH integrated analysis was performed on each.

#### PERCH Integrated Analysis Priors

The required starting values (priors) for etiology were noninformative (ie, did not favor any pathogen over others); the expected etiologic fraction for each pathogen plus the NoA category was equal to 7.7% (n = 1/13). The etiology prior distribution was set so that a few pathogens are responsible for most of the pneumonia (Dirichlet 0.3) (see **Supplementary Figure 2**). Sensitivity priors for NP/OP PCR were set to 60%–90% for each pathogen and for blood culture were set to 5%–25% for each pathogen (table in Figure 4A).

#### PERCH Integrated Analysis Results

The mean and selected quantiles (boxplot) of the distribution of the resulting 500 etiologic fraction estimates for each pathogen are shown in Figure 4A. In the ideal case with known sensitivities, the PERCH integrated analysis produced close to unbiased estimates for all pathogens, regardless of the type of data available or the magnitude of sensitivity and specificity of the measurements. Results for pathogens that had both bronze- and silver-standard measurements (B1, C1, and D1) were more frequently close to the true value compared with pathogens with only 1 measurement (B2–B3, C2–C3, and D2–D3), demonstrating value in combining evidence from multiple measurements, even when their individual results are similar. For example, B1 had both specimens available and the bias and standard deviation of the estimate was less than that observed when only bronze- (B2) or silver-standard (B3) data were available (**Supplementary Table 2**). When the bronze-standard data were uninformative (ie, OR, 1.0) as with pathogen B4, results were similar to cases with silver-standard data only (B3), thus showing no benefit of including these data but little harm either. The mean sensitivity for the silver-standard sensitivity prior range of 5%–25% is slightly lower than the true sensitivity of 15%, resulting in a minor overestimation for pathogens with silver-standard data only (B3, C3, and D3) or with uninformative bronze-standard data (B4). Note that pathogen E, a noncausative organism, received a low (<1%) but nonzero estimate as expected because all pathogens analyzed will have nonzero estimates. Figure 4B displays results from 1 of the simulated datasets that contributed to the distribution in Figure 4A in the form of a bubble plot. Pathogen A had the largest bubble as expected given it had the highest odds ratio. For each of the pathogens, the 95% CI of the etiology fraction estimate (denoted by whiskers) covered the true value.

##### Performance of the PERCH Integrated Analysis When Sensitivity Priors Are Inaccurate or Imprecise.

**Figure 5.**
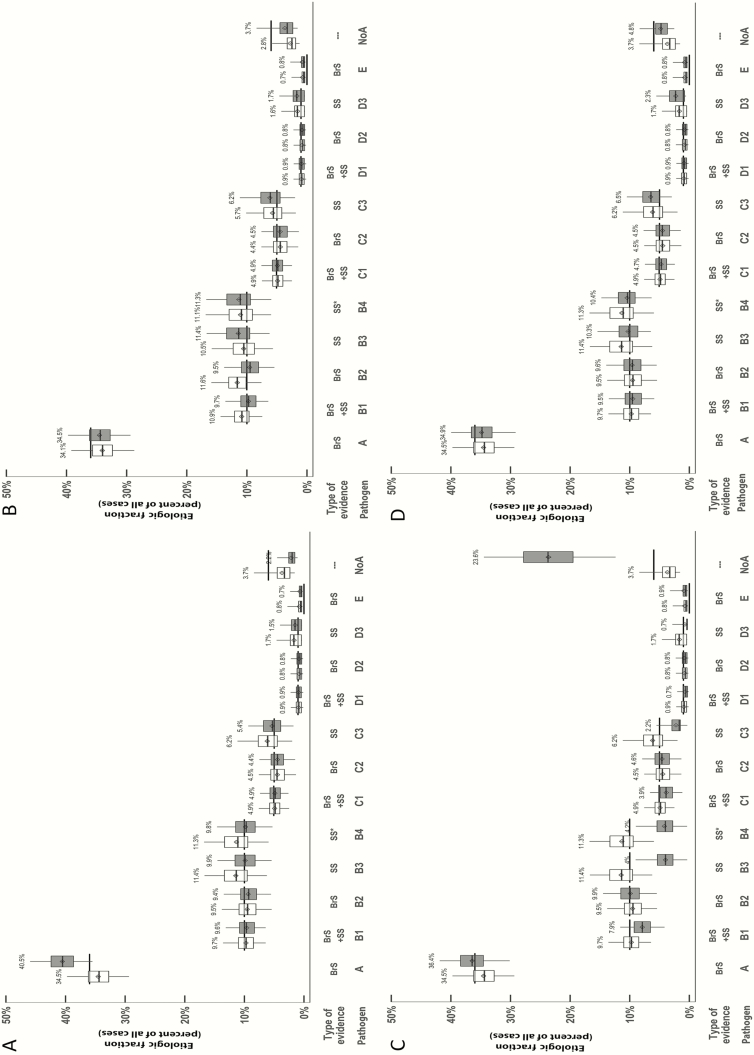
Impact of inaccurate or imprecise sensitivity priors on etiologic estimation from the PERCH integrated analysis using 500 simulated datasets: a sensitivity analysis compared with the base scenario presented in Figure 4. *A,* Impact of changing true bronze-standard (BrS) sensitivity for pathogen A from 75% (white) to 90% (gray). *B,* Impact of changing true bronze-standard (BrS) sensitivity for pathogen B1, B2, and B3 from 75% (white) to 90% (gray). *C,* Impact of changing true silver-standard (SS) sensitivity for pathogens B1, B3, B4, C1, C3, D1, and D3 from 15% (white) to 5% (gray) and changing SS sensitivity prior from 5%–25% (white) to 10%–20% (gray). *D,* Impact of increasing width of SS sensitivity prior from 5%–25% (white) to 1%–50% (gray). Description of boxplots: Bold black line, mean of the true value across the 500 datasets; Boxplots display the distribution of etiologic fraction point estimates from 500 simulated datasets: Diamond, average etiologic estimate across the 500 datasets; Vertical line through diamond, confidence interval around the average etiologic estimate; Numbers above boxplots indicate the numeric value of the diamond; whiskers denote the 5th and 95th percentiles of the etiologic fraction point estimates. Abbreviation: BrS, bronze-standard data (imperfect sensitivity and imperfect specificity; eg, nasopharyngeal polymerase chain reaction); NoA, none-of-the-above (ie, pathogens not tested for); SS, silver-standard data (imperfect sensitivity and perfect specificity; eg, blood culture).

 We evaluated 5 plausible scenarios for pneumonia etiology studies in which the assumptions of the prior sensitivities in the PERCH integrated analysis performed on the 500 simulated datasets described above and presented in Figure 4B were modified to assess the impact of inaccurate or imprecise sensitivity priors (Figure 5 and **Supplementary Table 3**). In general, specifying inaccurate or imprecise sensitivity priors had small to modest impact. One exception is when silver-standard sensitivity is substantially inconsistent with the true sensitivity.

*Scenario 1: Underestimating the true bronze-standard sensitivity for a pathogen with a large etiologic fraction that only has bronze-standard data.* In comparison with the data in Figure 4A, we changed the true sensitivity for the dominant pathogen A to 90% so that the 75% mean sensitivity prior (range, 60%–90%) was an underestimate; the scenarios for the remaining pathogens were left unchanged. The bias caused by this sensitivity prior was to overestimate the etiologic fraction of pathogen A by 5% on average (Figure 5A and **Supplementary Table 3*A***). Because the etiologic fractions estimated for all pathogens must sum to 100%, there is an indirect impact on the estimates for the other pathogens that are biased downward. The magnitude by which they will be affected will be a function of the strength of the evidence in their data. This can be observed for the NoA group, which represents pathogens not tested for and thus with no data. Its bias is greatest (estimate decreased from the true value of 6% to 2.2%), whereas the estimates for the other pathogens with data were only minimally impacted.

*Scenario 2: Underestimating the true bronze-standard sensitivity for pathogens with and without silver-standard data.* To further examine the effects of underestimating bronze-standard sensitivity priors, we changed the true sensitivity to 90% for 3 pathogens, each with 10% true etiology, while leaving the mean sensitivity prior at 75% (range, 60%–90%). Compared with the results in Figure 4A, etiology was only slightly overestimated for the 2 pathogens (B1–B2) with moderate bronze-standard data (OR, 2), but pathogen B1, which also had silver-standard data, was less affected (average bias was 0.9% vs 1.6%; Figure 5B and **Supplementary Table 3*B***). The effects on pathogens without informative bronze-standard data (ie, OR, 1 for silver-standard only data; B3–B4) were minimal and only due to the indirect effect in response to the increase in B2. The cumulative mean bias again mostly affected the NoA group, but pathogen A also had a slight (0.5%) mean decrease relative to the unbiased analysis in Figure 4A.

*Scenario 3: Overestimating the true silver-standard sensitivity.* To evaluate impact of mis-specifying silver-standard sensitivity priors, we changed the true sensitivity to 5% for all pathogens with silver-standard data and changed the sensitivity priors to exclude the true value (range, 10%–20%). The etiology estimates were underestimated for all pathogens with silver-standard data, with a substantial cumulative bias of approximately 21%, and perturbations in the rank order of the pathogens, thus demonstrating the importance of the silver-standard sensitivity priors in the final estimates (Figure 5C and **SupplementalryTable 3*C***). The largest impact was on pathogens with the most silver-standard data and uninformative bronze-standard data (B3 and B4), biasing their true etiologic fraction from 10% to approximately 4%. The cumulative bias was almost exclusively reflected as a 4-fold increase in the NoA group, with virtually no indirect effects on etiology for pathogens without silver-standard data, including the noncausative pathogen E, which remained near zero, demonstrating that evidence against causality is still evidence.

*Scenario 4: Increasing the range of the silver-standard sensitivity prior.* To evaluate the effect of wide silver-standard sensitivity priors but where both still covered the true sensitivity of 15%, we widened ranges from 5%–25% in Figure 4A to 1%–50% in Figure 5D. Widening the sensitivity prior range had little effect on the etiologic faction estimates as these changed ≤1% for all pathogens and the rank order of the pathogens was preserved (Figure 5D). But widening the sensitivity priors produced greater uncertainty in the estimates for pathogens with only silver-standard evidence (ie, 5%–6% wider 95% CIs for pathogens B3, B4, C3, and D3) (**Supplementary Tables 2** and **3*B*** and **Supplementary Figure 4**).

*Scenario 5: Measurements with poorer bronze-standard specificity.* Although the bronze-standard specificities selected for the above simulations generally reflect those observed in the PERCH study for most measurements, we examined how the PERCH integrated analysis performed when specificity decreased from >90% in Figure 4A to 75% (odds ratios changed from 2.0 to 1.0–1.3 for most pathogens; **Supplementary Figure 5**). The impact on etiologic faction estimates was minimal (<2% change for all pathogens), primarily limited to pathogens with bronze-standard–only data, and there was no impact on rank order (**Supplementary Figure 5*B***). But the 95% CIs widened 2%–6% for all pathogens, not just those with bronze-standard data (**Supplementary Figures 5*C*** and **5*D***), increasing the uncertainty of the results overall.

## LIMITATIONS AND OPPORTUNITIES OF THE PERCH INTEGRATED ANALYSIS

Despite the utility of this approach to expand the analytic options to enable integration of data from multiple, imperfect measurements, like any statistical model, the PERCH integrated analysis has limitations. Although the current version of the PERCH integrated analysis is flexible enough to meet most of the challenges of analyzing pneumonia etiology data, the PERCH integrated analysis cannot solve or even address the biggest problem in determining pneumonia etiology, which is that most of the evidence comes from peripheral sources and not directly from the site of infection. Even peripheral measurements with perfect specificity (ie, blood culture) have low prevalence and can provide information only for bacterial pathogens, not viruses. The strength of the evidence and the robustness of the inferences from the PERCH integrated analysis would be greater with more silver-standard blood culture– and lung aspirate–confirmed cases, which both reduces uncertainty around these measures and better informs on the sensitivity of the silver- and bronze-standard NP/OP PCR data. Obviously, this inability to quantify or correct for systematic bias in the peripheral measurements applies to any analysis of pneumonia etiology.

An essential assumption of the PERCH integrated analysis is that, when a pathogen is the cause of pneumonia, it is the only pathogen whose chance of being measured in the periphery is increased by its infection of the lung. This assumes that the peripheral measurements are unbiased reflections of the state of the lung. If instead, infection by 1 pathogen in the lung increases the presence of other pathogens in the periphery, the PERCH integrated analysis and any other analysis of the peripheral data alone will be biased, absent external knowledge that could be used for correction. In addition, in situations where >1 pathogen is determined to be in the lung, case–control studies by design cannot determine whether only 1 or more than 1 are the cause.

A limitation of any Bayesian analysis is that the prior distributions for etiology, sensitivity, and specificity are ultimately reliant on the state of knowledge at the time. Despite the fact that the priors are objectively updated by data, this raises the question of the degree to which the resulting posterior distributions are influenced by the prior assumptions, rather than just the data. This is mitigated in that Bayesian methods are flexible enough to incorporate our uncertainty about unknowns and we can perform sensitivity analyses to determine which assumptions are most influential on the substantive findings; when changing the prior assumptions within a plausible class does not meaningfully change the results, we can be more assured that the data are largely determining the outputs by comparison to the priors.

Currently the PERCH integrated analysis is tailored for binary (positive/negative) test results so that continuous data such as pathogen density must be categorized into above and below a threshold. Although continuous measurements are becoming more of interest in diagnostics, most traditional test results are still binary, and implementing thresholds enables utilization of continuous data. However, extending the model to include categorized data (eg, low, medium, high) is relatively easy and may further improve estimation.

The PERCH integrated analysis has recently been upgraded to enable regression-like adjustments to account for factors associated with pathogen distribution, such as seasonality and age, and to produce stratified output when etiology might differ depending on case conditions, such as pneumonia severity or chest x-ray positivity. However, adjustment for multiple variables or continuous variables currently makes the computing time prohibitive. But the method easily accommodates categorical covariates, and analyses can be indirectly adjusted for an additional covariate by stratifying and recombining using a standard population.

## SOFTWARE

The Bayesian method for estimating population and individual etiology fractions is implemented by connecting 2 freely available software programs in tandem on Linux or Windows operating systems: statistical computing language R-3.3.1 (http://cran.r-project.org/) [12] and Bayesian inference software JAGS 4.2.0 (http://mcmc-jags.sourceforge.net/) [13]. The PERCH integrated analysis combines these tools into an analytic pipeline for etiology research in a publicly available R package (https://github.com/zhenkewu/baker).

The R package, named the Bayesian Analysis Kit for Etiology Research, implements both exploratory and model-based analyses of data collected for etiology research. The package enables an analyst to organize diagnostic test results by their measurement standards (bronze, silver, and gold standard) and to calculate summaries such as the pathogen-specific positive rates for cases and controls along with their comparison by the odds ratio. The analyst can then specify which test results to integrate, which pathogens to include, and their sensitivity and etiology priors, among other model components. Based on these model specifications, R calls and instructs JAGS to fit the corresponding model to the selected data, performs model diagnostics, and stores the posterior results for ensuing inference of the key unknown parameters, such as the population and individual etiology distributions. Finally, the package offers optional visuals as an aid to displaying the evidence in the data for disease etiology and to facilitate model criticism.

## DISCUSSION

Pneumonia etiology studies in the current era commonly assess NP/OP PCR data in cases and controls and blood culture results in cases, among other evidence. Until now, we have been unable to integrate these results and their sensitivity and specificity in a systematic, quantitative way to estimate the etiologic fractions for a set of pathogens given a population of cases. Here we have described and demonstrated the performance of an open-source available method that has been developed to integrate such data for etiology research. Although attributable fraction and other analytic approaches to assess pneumonia etiology such as expert review of case-only results, case-control logistic regression, and latent class analysis have served well in many disease conditions and produced valuable insights, modern studies of pneumonia etiology call for an expanded approach. We developed the PERCH integrated analysis method, which builds on attributable fraction and latent class analysis approaches, to enable analyses of PERCH data that integrate results from multiple tests from multiple specimens to assess the cause of pneumonia, while accounting for their imperfect sensitivity and specificity.

The PERCH integrated analysis method builds on and extends previous familiar analytic methods such as latent class analysis and attributable fraction that are applicable for simpler conditions when only 1 pathogen is being evaluated or only 1 specimen is collected from cases and controls, respectively, but which are not directly applicable for pneumonia study conditions [1]. The PERCH study assesses >30 pathogens detected using 2–3 types of tests on 2–4 specimens collected from each case plus 2 specimens collected from each control. The PERCH integrated analysis method is able to integrate all of the PERCH data and, with informative priors, data from other studies to produce synthesized estimates.

Bayesian methods used by the PERCH integrated analysis force us to recognize the role of such often ignored parameters. All methods make prior assumptions regarding these parameters; some do not explicitly specify prior uncertainty about them.

PERCH data have substantial limitations. In simplest terms, we seek to know the frequency with which pathogens infect lungs, and PERCH cannot measure the lung (with a few exceptions), only the blood and NP/OP. A similar state of affairs exists in many sciences: particle physics (particle traces in steam), astronomy (waves impinging on telescopes), and chemistry (colorimetry), to name a few. Although Bayesian methods present a solution, they also present a challenge in that they are not as well understood or as widely used as frequentist statistics in the international health community and may be mistrusted as nontransparent “black box” methods. Nevertheless, the reality is that, far from being new, this approach has been successfully used in many scientific applications since Thomas Bayes introduced it 250 years ago. One of its most notable applications was by Alan Turing to crack the enigma code [14]. The PERCH integrated analysis may be likened in that respect to a modern internal-combustion-engine car: its core (engine, wheels, controls) is simple, not different than was used 100 years ago, but there are lots of modern “bells and whistles,” each of which improves its performance. The availability of open-source software to conduct the PERCH integrated analysis will enable wider use of Bayesian tools. This paper intends to make the Bayesian methods transparent in its functions, removing the perception of black box analyses in the pneumonia community.

The analysis presented here assumes a single-pathogen cause model, but it can accommodate copathogens by assigning prescribed combinations of pathogens. We are exploring methods to expand the PERCH integrated analysis to enable it to evaluate all possible combinations of pathogens while favoring more parsimonious etiologies.

It is important to reiterate that any statistical analysis can at best provide a valid summary of the evidence and remaining uncertainties about disease etiology. The most informative advancement for pneumonia etiology studies will come from addressing the limitations of the data, in particular the lack of measurements that are both moderately sensitive and specific. Measurement limitation remains the greatest barrier to precisely determining etiology, whether caused by only 1 or >1 pathogen.

Finally, pneumonia etiology studies are not unique in needing methods to integrate multiple measurements. For example, the Aetiology of Neonatal Infections in South Asia (ANISA) case–control study aims to determine the etiology of serious neonatal infections, including sepsis and meningitis [15]. The ANISA study needs to integrate multiple test results from blood, cerebrospinal fluid, and NP/OP specimens collected from cases with those from blood and NP/OP specimens collected from controls. This suggests that the PERCH integrated analysis has applications beyond pneumonia etiology research.

## CONCLUSIONS

The existing methods to estimate pneumonia etiology, including attributable fraction, latent class analysis, case–control odds ratio estimation, and expert panel reviews of case-only data, are not able to integrate data from multiple specimen types, multiple measurement types, and for multiple pathogens while also accounting for measurement error. The PERCH integrated analysis is a novel analytic method that surmounts these limitations by integrating evidence from multiple, imperfect diagnostic measurements on cases and controls to estimate the etiologic distribution and assign etiology to individuals, all with estimations of uncertainty for the population of cases and for each individual case. This analytic advance has potential applications in etiology research beyond pneumonia.

## Supplementary Data

Supplementary materials are available at *Clinical Infectious Diseases* online. Consisting of data provided by the authors to benefit the reader, the posted materials are not copyedited and are the sole responsibility of the authors, so questions or comments should be addressed to the corresponding author.

## Supplementary Material

Supplemental Tables and Figures

## Notes

***Author contributions.*** M. D. K. drafted the manuscript, designed the simulation analysis plan for the paper, interpreted results and provided epidemiologic expertise to the analytic method development and application to PERCH. S. L. Z. and Z. W. developed the analytic methods and assisted with the simulation analysis plan, interpretation of results, and drafting the manuscript. L. L. H., K. L. O., D. R. F., J. A. G. S., and O. S. L. provided clinical and epidemiologic expertise to the development of the analytic method. L. L. H., K. L. O., D. R. F., H. C. B., S. R. C. H., J. A. G. S., C. P., and O. S. L. assisted with drafting of the manuscript. L. L. H., K. L. O., D. R. F., H. C. B., S. R. C. H., J. A. G. S., S. A. M., D. M. T., W. A. B., and K. L. K. provided clinical and epidemiologic guidance on adaption of the analysis and application of the analysis to the PERCH setting. Q. S., W. F., and M .L. contributed to the design of the analytic methods. Q. S., W. F., and W. L. performed the simulation analyses. C. P., W. F., M. L., and D. E. P. coordinated the development of the analytic methods and the conduct of the simulation analyses and contributed to the writing.

***Acknowledgments.*** The authors would also like to recognize William C. Blackwelder, a member of the PERCH Pneumonia Expert Group, for providing insights into attributable fraction analysis methods. This paper is published with the permission of the Director of the Kenya Medical Research Institute.

***Financial support.*** PERCH was supported by grant 48968 from the Bill & Melinda Gates Foundation to the International Vaccine Access Center, Department of International Health, Johns Hopkins Bloomberg School of Public Health. J. A. G. S. was supported by a clinical fellowship from The Wellcome Trust of Great Britain (098532).

***Supplement sponsorship.*** This article appears as part of the supplement “Pneumonia Etiology Research for Child Health (PERCH): Foundational Basis for the Primary Etiology Results,” sponsored by a grant from the Bill & Melinda Gates Foundation to the PERCH study of Johns Hopkins Bloomberg School of Public Health, Baltimore, Maryland.

***Potential conﬂicts of interest.*** M. D. K. has received funding for consultancies from Merck, Pfizer, Novartis, and grant funding from Merck. L. L. H. has received grant funding from Pfizer and GlaxoSmithKline. K. L. K. has received grant funding from Merck Sharp & Dohme. S. A. M. has received honorarium for advisory board from Bill & Melinda Gates Foundation (BMGF), Pfizer, Medimmune, and Novartis, institutional grants from GSK, Novartis, Pfizer, Minervax, and BMGF, and speakers bureau for Sanofi Pasteur and GSK. K. L. O. has received grant funding from GSK and Pfizer and participates on technical advisory boards for Merck, Sanofi Pasteur, PATH, Affinivax, and ClearPath. All other authors: No reported conflicts. All authors have submitted the ICMJE Form for Disclosure of Potential Conflicts of Interest. Conflicts that the editors consider relevant to the content of the manuscript have been disclosed.
