# Supplementary material for: Bayesian Estimation of Pneumonia Etiology: Epidemiologic Considerations and Applications to the Pneumonia Etiology Research for Child Health Study
Source: Clin Infect Dis. 2017 May 27;64(Suppl 3):S213–27. doi: 10.1093/cid/cix144 (PMC5447849; doi:10.1093/cid/cix144)

Supplemental Table 1. Etiologic fraction estimates presented in Figure 3 comparing results from attributable fraction (AF) and PERCH Integrated Analysis (PIA): output averaged across 500 simulated datasets

Pathogen	Truth (%)	Etiologic Fraction Estimate[a] (95% CI)		Bias[b]		Standard Deviation[c]		RMSE[d]		Coverage[e]		95% CI Length[f]	
		AF	PIA	AF	PIA	AF	PIA	AF	PIA	AF	PIA	AF	PIA
A	30	31.3 (26.2, 36.3)	29.7 (24.6, 34.7)	1.3	-0.36	2.6	2.6	2.9	2.6	97.2	99.2	10.2	10.0
B	30	34.9 (25.6, 43.5)	29.5 (22.3, 36.5)	4.97	-0.42	4.5	3.4	6.7	3.4	82.4	99.0	17.9	14.2
C	15	12.4 (7.4, 17.3)	14.9 (9.4, 20.7)	-2.57	-0.09	2.6	2.9	3.6	2.9	84.6	98.2	9.9	11.3
D	15	9.5 (1.4, 19.6)	14.0 (3.8, 24.9)	-5.55	-0.98	5.0	4.8	7.5	4.9	79.8	97.8	18.2	21.1
NoA	10	13.4 (1.8, 29.3)	11.9 (2.2, 24.1)	3.4	1.84	7.4	4.7	8.2	5.0	94.4	98.6	27.5	21.9

Abbreviations: PIA_N, results from PIA method using narrow sensitivity priors; PIA_W, results from PIA method using wide sensitivity priors; CI, credible interval; RSME, root mean square error.

[a] Estimate is defined as the mean etiologic estimate across the 500 datasets.
[b] Bias is defined as the difference between the mean etiologic estimate and the true etiologic fraction values used to generate the data sets.
[c] Standard deviation is defined as the square root of the variance across the 500 datasets.
[d] Root mean square error (RMSE) is a measure of error, incorporating both bias and variance. RMSE is calculated as the average of the squared differences between the mean etiologic estimate and the truth for each of the 500 datasets.
[e] Coverage is defined as the observed fraction of the 95% credible intervals that cover the true etiology fractions out of the 500 replications.
[f] Average length of the 95% credible intervals across the 500 datasets.

Supplemental Figure 1. Etiologic fraction estimates using attributable fraction (AF) and PERCH Integrated Analysis (PIA), where AF calculated using two methods.

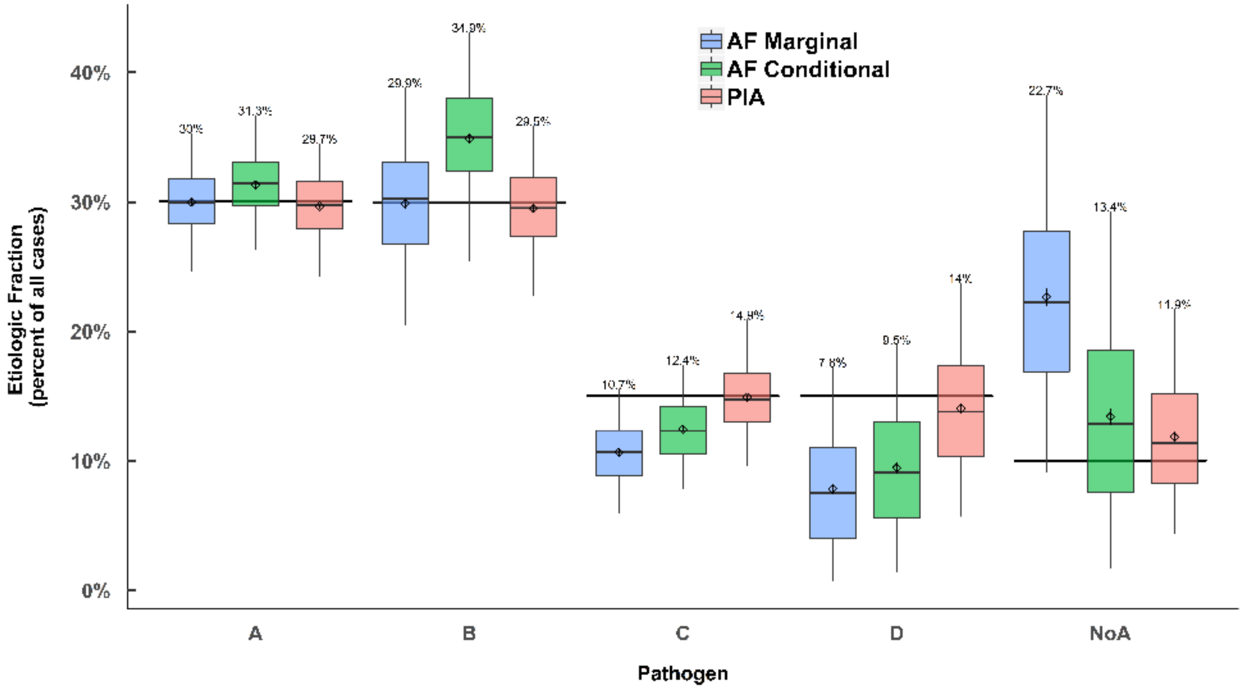

Legend:
Distribution of the output from 500 simulated datasets by analysis method. AF Marginal calculated using odds ratios obtained from a logistic regression where only the single pathogen is used to predict case-control status. AF Conditional calculated using odds ratios obtained from a multi-variable logistic regression where the pathogen and all others predict case-control status.

Description of boxplots: Bold black line = mean of the true value across the 500 datasets; Boxplots display the distribution of etiologic fraction point estimates from 500 simulated datasets: Diamond = average etiologic estimates across the 500 datasets; Vertical line through diamond = confidence interval around the etiologic estimates; Numbers above boxplots indicate the numeric value of the diamond; whiskers denote the 5th and 95th percentiles of the etiologic fraction point estimates.

Supplemental Figure 2: Dirichlet etiology priors for 30 etiology fractions (pathogens) at three hyperparameter values, alpha=0.1 (blue), 0.5 (green), 1(red).

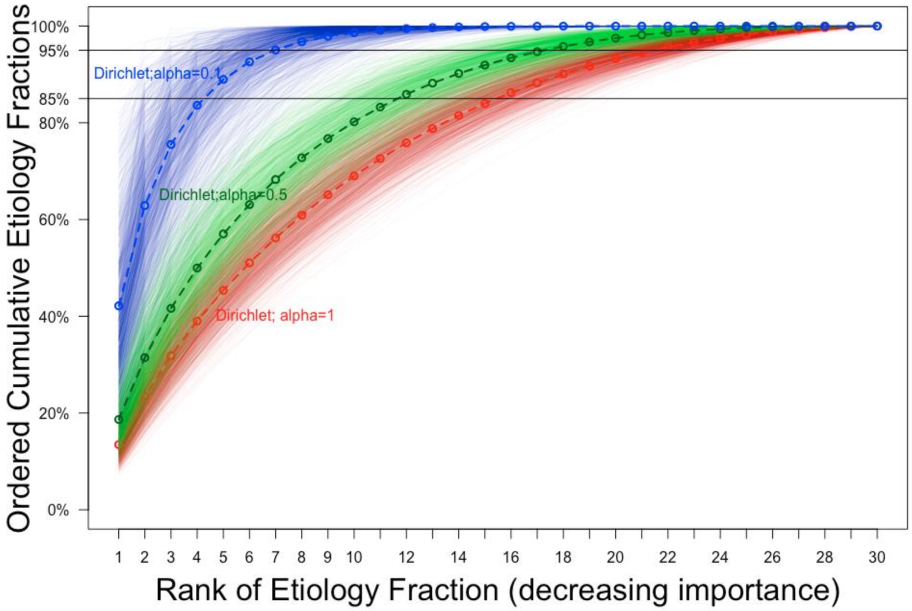

Legend: Given alpha, 1,000 random realizations from the prior are shown, each by a light curve of identical color; the dotted curve lying at the center of the light curves, from left to right with decreasing etiologic importance, characterizes the average cumulative etiology fractions *in a priori*.

Bayesian analysis uses the evidence in the data to calculate a posterior distribution for the unknowns from an input prior distribution. Because the goal of this analysis is to elucidate the evidence in the case-control data about the unknown etiology fractions, we have used minimally informative priors so that the posterior mainly represents the PERCH evidence. Specifically, we use a Dirichlet prior distribution (extension to multiple pathogens of the uniform distribution) that assumes that: (1) every pathogen or pathogen combination is equally likely to be the cause for a given child; and (2) that roughly 85% of the cases will be caused by the 5 top pathogens. When using regression models to adjust for covariates, we use a (logistic-normal) prior distribution for the regression parameters that closely matches this Dirichlet.

The blue curve shows that*, in a priori*, a Dirichlet prior with alpha=0.1 encourages a few large etiology fractions, leaving not much room for smaller ones. This is evident from the fast plateaued cumulative curve for the largest, 2nd largest, 3rd largest, ... etiology fractions. We show 1,000 realizations from this prior, each by a light blue curve. On average (blue dashed lines), roughly 85% of all the cases are caused by 4 top pathogens; 95% of all the cases are caused by top 7 pathogens.

Increasing alpha from 0.1 to 0.5 and 1 (blue, to green and red) admits tiny etiology fractions *in a priori*. This is evident from the less steep cumulative curves (green for 0.5; red for 1). A Dirichlet prior with alpha=0.5 assumes 17 top pathogens will fill 95% of the entire pie on average, and 21 pathogens when alpha=1.

Supplemental Figure 3: Etiologic fraction estimates using PERCH Integrated Analysis (PIA) comparing narrow and wide sensitivity priors performed on 500 simulated datasets for a study that had only one specimen collected from 600 cases and 600 controls.

a. True values for 500 simulated datasets and input parameter values

	Pathogen				
**Parameter**	A	B	C	D	NoA
**True Etiology (%)**	30	30	15	15	10
**Sensitivity (%)**	90	90	75	75	NA
**Specificity (%)**	85	50	85	50	NA
**Cases testing positive[a] (%)**	37.6	62.1	24.0	53.8	NA
**Controls testing positive[a] (%)**	15.1	50.0	14.9	50.2	NA
**Odds Ratio[a]**	3.4	1.7	1.8	1.2	NA
**Narrow Sensitivity Prior (%)**	89.55-90.45	89.55-90.45	74.99-75.01	74.99-75.01	NA
**Wide Sensitivity Prior (%)**	73-99	73-99	56-90	56-90	NA

NA, Not applicable [None-of-the-above pathogens (NoA) reflects pathogen(s) not tested for so input parameters are not applicable].
[a]Prevalence and odds ratios estimated by averaging across the values within the 500 datasets that were created based on the true etiology, sensitivity and specificity values.

b. Distribution of etiologic estimates from PIA analyses of 500 simulated datasets, analyzed using narrow versus wide sensitivity priors

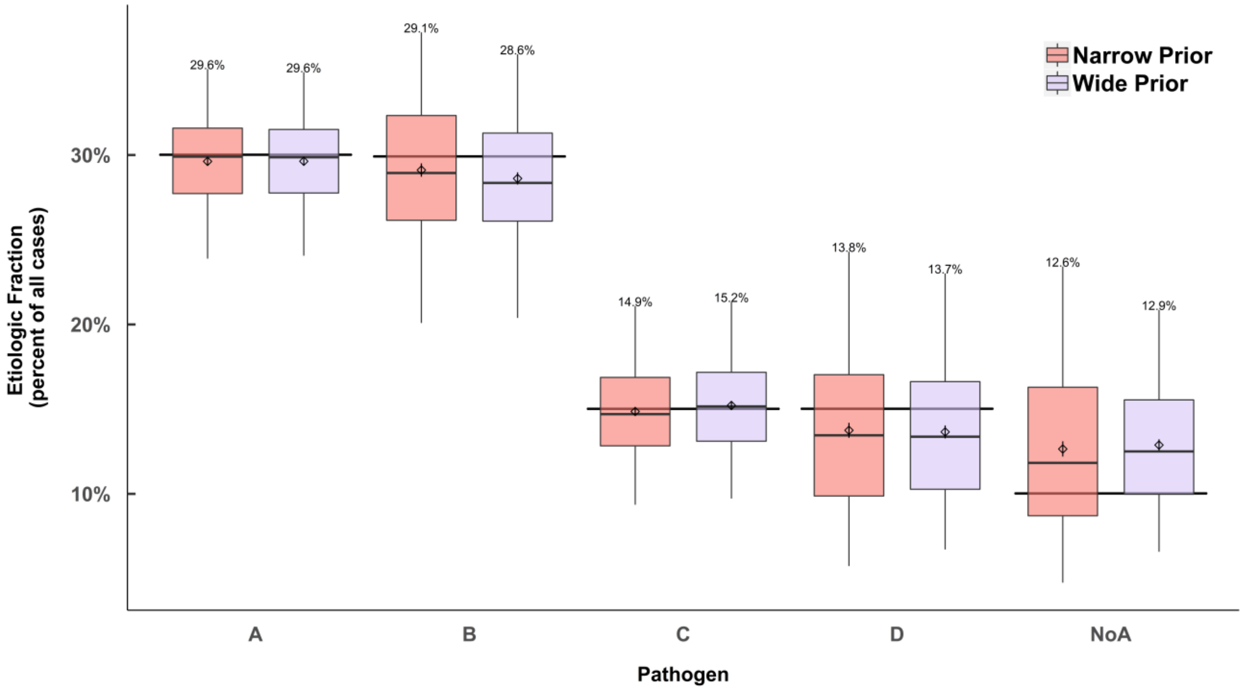

Bold black line = mean of the true value across the 500 datasets; Boxplots display the distribution of etiologic fraction point estimates from 500 simulated datasets: Diamond = average etiologic estimates across the 500 datasets; Vertical line through diamond = confidence interval around the etiologic estimates; Numbers above boxplots indicate the numeric value of the diamond; whiskers denote the 5th and 95th percentiles of the etiologic fraction point estimates.

c. PIA Method, Comparing Narrow and Wide Sensitivity Priors

Pathogen	Truth (%)	Estimate[a] (95% CI)		Bias[b]		Standard Deviation[c]		RMSE[d]		Coverage[e]		95% CI Length[f]	
		PIA_W	PIA_N	PIA_W	PIA_N	PIA_W	PIA_N	PIA_W	PIA_N	PIA_W	PIA_N	PIA_W	PIA_N
A	30	29.6 (22.9,38.8)	29.6 (24,35.3)	-0.39	-0.39	2.9	3	2.9	3.0	100	98.4	15.8	11.3
B	30	28.6 (18,42.2)	29.1 (19.6,38.2)	-1.3	-0.81	4.1	4.5	4.3	4.6	99.8	97.4	24.2	18.6
C	15	15.2 (8.6,24)	14.9 (9,20.9)	0.2	-0.15	3.1	3.1	3.1	3.1	99.4	97.4	15.5	11.9
D	15	13.7 (2.6,28.6)	13.8 (3.1,25.5)	-1.37	-1.27	4.4	5	4.6	5.2	99.8	98.8	25.9	22.4
NoA	10	12.9 (0.9,29.2)	12.6 (2,26.8)	2.86	2.63	3.8	5.1	4.8	5.8	100	98.6	28.3	24.8

Abbreviations: PIA_N, results from PIA analysis, using narrow sensitivity priors; PIA_W, results from PIA analysis, using wide sensitivity priors; CI, credible interval; RSME, root mean square error.

[a] Estimate is defined as the mean etiologic estimate across the 500 datasets.
[b] Bias is defined as the difference between the mean etiologic estimate and the true etiologic fraction values used to generate the data sets.
[c] Standard deviation is defined as the square root of the variance across the 500 datasets.
[d] Root mean square error (RMSE) is a measure of error, incorporating both bias and variance. RMSE is calculated as the average of the squared differences between the mean etiologic estimate and the truth for each of the 500 datasets.
[e] Coverage is defined as the observed fraction of the 95% credible intervals that cover the true etiology fractions out of the 500 replications.
[f] Average length of the 95% credible intervals across the 500 datasets.

Supplemental Table 2. Output for simulation in Figure 4 presenting mean etiologic estimate, bias, SD, coverage, etc.

	Truth (%)	Estimate[a] (95% CI)	Bias[b]	Standard Deviation[c]	RMSE[d]	Coverage[e]	95% CI Length[f]
A	36	34.5 (27.2, 44)	-1.4	2.7	3	100	16.8
B1	10	9.7 (5.9, 14.3)	-0.3	1.9	1.9	99.6	8.3
B2	10	9.5 (5.3, 14.7)	-0.5	2.2	2.3	98.4	9.5
B3	10	11.4 (4.3, 22.3)	1.4	2.9	3.1	99.4	17.9
B4	10	11.3 (4.3, 22)	1.3	2.8	3.2	100	17.6
C1	5	4.9 (2.4, 8)	-0.1	1.3	1.3	99.6	5.6
C2	5	4.5 (1.6, 7.9)	-0.5	1.6	1.7	97.4	6.3
C3	5	6.2 (1.6, 14.8)	1.2	2.4	2.7	98.8	13.1
D1	1	0.9 (0.2, 2.3)	-0.1	0.6	0.6	97.4	2.2
D2	1	0.8 (0.1, 2.3)	-0.2	0.6	0.6	98.4	2.3
D3	1	1.7 (0.2, 6)	0.7	1.3	1.5	98.6	5.9
E	0	0.8 (0, 3.2)	0.8	0.6	1	0.6	3.2
NoA	6	3.7 (0, 15.9)	-2.3	1.9	2.9	100	15.9

Abbreviations: CI, credible interval; RSME, root mean square error.

a. Estimate is defined as the mean etiologic estimate across the 500 datasets.
b. Bias is defined as the difference between the mean etiologic estimate and the true etiologic fraction values used to generate the data sets.
c. Standard deviation is defined as the square root of the variance across the 500 datasets.
d. Root mean square error (RMSE) is a measure of error, incorporating both bias and variance. RMSE is calculated as the average of the squared differences between the mean etiologic estimate and the truth for each of the 500 datasets.
e. Coverage is defined as the observed fraction of the 95% credible intervals that cover the true etiology fractions out of the 500 replications.
f. Average length of the 95% credible intervals across the 500 datasets.

Supplemental Table 3: Impact of inaccurate or imprecise sensitivity priors on etiologic estimation from PERCH Integrated Analysis using 500 simulated datasets: output results

a. Underestimating Bronze-standard (BrS) sensitivity for a domnant pathogen (pathogen A)

From the boxplot for Pathogen A in Figure 4a it may appear that the results very rarely cover the truth, but the coverage shown here is 98% indicating that 98% of the 500 analyses had results whose confidence interval covered the true value. This makes sense if you understand the boxplot in Figure 4a is presenting the distribution of the etiologic *point* estimates across the 500 analyses whereas the coverage value describes how often the 95% CI from any single analysis covers the truth. So the etiology estimate is very rarely in line with the truth of 36% (across the 500 datasets), but even when it's way off base (e.g., 43%) then 95% CI still covers the truth because of the length of the CI.

	Truth (%)	Estimate[a] (95% CI)	Bias[b]	Standard Deviation[c]	RMSE[d]	Coverage[e]	95% CI Length[f]
A	36	40.5 (33.2, 49.5)	4.6	2.7	5.3	98	16.3
B1	10	9.6 (5.9, 13.9)	-0.4	1.7	1.8	99.6	8
B2	10	9.4 (5.4, 14.2)	-0.6	2.1	2.1	98.2	8.9
B3	10	9.9 (4, 18.7)	-0.1	2.4	2.4	99	14.8
B4	10	9.8 (3.9, 18.5)	-0.2	2.4	2.4	99.6	14.6
C1	5	4.9 (2.4, 7.9)	-0.2	1.2	1.3	99.8	5.5
C2	5	4.4 (1.7, 7.8)	-0.6	1.5	1.6	97.6	6
C3	5	5.4 (1.5, 12.3)	0.4	2	2.1	99.2	10.8
D1	1	0.9 (0.2, 2.3)	-0.1	0.6	0.6	97.4	2.1
D2	1	0.8 (0.1, 2.3)	-0.1	0.6	0.6	99	2.2
D3	1	1.5 (0.2, 5.2)	0.5	1.2	1.3	98.8	5.1
E	0	0.7 (0, 2.9)	0.7	0.5	0.9	0.6	2.9
NoA	6	2.2 (0, 11)	-3.8	1	3.9	96.6	11

b. Underestimating BrS sensitivity for common (10%) etiology pathogens (pathogens B1, B2 and B4)

	Truth (%)	Estimate[a] (95% CI)	Bias[b]	Standard Deviation[c]	RMSE[d]	Coverage[e]	95% CI Length[f]
A	36	34.1 (27.1, 42.9)	-1.9	2.6	3.3	100	15.8
B1	10	10.9 (7, 15.3)	0.9	1.8	2	98	8.3
B2	10	11.6 (7.2, 17.1)	1.6	2.2	2.7	97.8	9.9
B3	10	10.5 (4.1, 20.2)	0.5	2.6	3	99.2	16
B4	10	11.1 (4.4, 20.7)	1	2.8	2.7	100	16.4
C1	5	4.9 (2.4, 8)	-0.2	1.2	1.3	99.6	5.6
C2	5	4.4 (1.7, 7.9)	-0.6	1.6	1.7	97.4	6.2
C3	5	5.7 (1.6, 13.4)	0.7	2.2	2.3	98.8	11.9
D1	1	0.9 (0.2, 2.3)	-0.1	0.6	0.6	96.8	2.2
D2	1	0.8 (0.1, 2.3)	-0.2	0.6	0.6	98.8	2.3
D3	1	1.6 (0.2, 5.6)	0.6	1.2	1.4	98.6	5.4
E	0	0.7 (0, 3.1)	0.7	0.6	0.9	0.6	3.1
NoA	6	2.8 (0, 13.1)	-3.2	1.3	3.5	99.4	13.1

c. Overestimating Silver-standard (SS) sensitivity for all pathogens with SS data (pathogens B1, B3, B4, C1, C3, D1, D3)

	Truth (%)	Estimate[a] (95% CI)	Bias[b]	Standard Deviation[c]	RMSE[d]	Coverage[e]	95% CI Length[f]
A	36	36.4 (27.72,50.31)	0.46	3.1	3.1	100	22.59
B1	10	7.9 (4.02,12.37)	-2.09	1.97	2.9	88.4	8.36
B2	10	9.9 (5.36,15.61)	-0.11	2.41	2.4	98.8	10.24
B3	10	4.2 (1.03,10.13)	-5.81	2.08	6.2	49.2	9.1
B4	10	4.0 (0.98,9.79)	-5.98	2.18	6.4	46	8.8
C1	5	3.9 (1.36,7.01)	-1.19	1.43	1.9	93.8	5.65
C2	5	4.6 (1.61,8.23)	-0.42	1.71	1.8	97	6.62
C3	5	2.2 (0.35,6.5)	-2.76	1.48	3.1	70.8	6.15
D1	1	0.7 (0.06,2.11)	-0.27	0.53	0.6	95.2	2.05
D2	1	0.8 (0.05,2.39)	-0.14	0.6	0.6	97.4	2.34
D3	1	0.7 (0.04,3.28)	-0.25	0.74	0.8	99.6	3.24
E	0	0.9 (0,3.59)	0.91	0.78	1.2	0.6	3.59
NoA	6	23.6 (6.17,37.31)	17.66	5.71	18.6	56.6	31.14

d. Increasing range of SS sensitivity prior for all pathogens with SS data (pathogens B1, B3, B4, C1, C3, D1, D3)

	Truth (%)	Estimate[a] (95% CI)	Bias[b]	Standard Deviation[c]	RMSE[d]	Coverage[e]	95% CI Length[f]
A	36	34.9 (27.3,45.3)	-1.1	2.7	2.9	100.0	18.0
B1	10	9.5 (5.5,14.2)	-0.5	2.0	2.0	99.0	8.7
B2	10	9.6 (5.3,14.9)	-0.4	2.2	2.3	99.0	9.6
B3	10	10.4 (2.4,25.7)	0.4	2.2	2.2	100.0	23.2
B4	10	10.3 (2.5,25.0)	0.3	2.4	2.4	100.0	22.5
C1	5	4.7 (2.1,7.9)	-0.3	1.3	1.3	99.6	5.8
C2	5	4.5 (1.6,8.0)	-0.5	1.6	1.7	97.2	6.4
C3	5	6.5 (1.0,20.4)	1.5	2.0	2.5	99.8	19.4
D1	1	0.9 (0.13,2.3)	-0.1	0.6	0.6	97.2	2.2
D2	1	0.8 (0.05,2.4)	-0.1	0.6	0.6	98.6	2.3
D3	1	2.3 (0.12,11.2)	1.3	1.5	2.0	99.8	11.1
E	0	0.8 (0.0,3.3)	0.8	0.6	1.0	0.0	3.3
NoA	6	4.8 (0.0,20.9)	-1.1	1.5	1.9	100.0	20.9

Abbreviations: CI, credible interval; RSME, root mean square error.

a. Estimate is defined as the mean etiologic estimate across the 500 datasets.
b. Bias is defined as the difference between the mean etiologic estimate and the true etiologic fraction values used to generate the data sets.
c. Standard deviation is defined as the square root of the variance across the 500 datasets.
d. Root mean square error (RMSE) is a measure of error, incorporating both bias and variance. RMSE is calculated as the average of the squared differences between the mean etiologic estimate and the truth for each of the 500 datasets.
e. Coverage is defined as the observed fraction of the 95% credible intervals that cover the true etiology fractions out of the 500 replications.
f. Average length of the 95% credible intervals across the 500 datasets.

Supplemental Figure 4: Impact of imprecise sensitivity priors on etiologic estimation from PERCH Integrated Analysis: Bubble plot results from PIA analysis of a randomly selected dataset from (**Supplemental Table 3d**) above, evaluating a wider SS sensitivity prior range for all pathogens with SS data (pathogens B1, B3, B4, C1, C3, D1, D3)

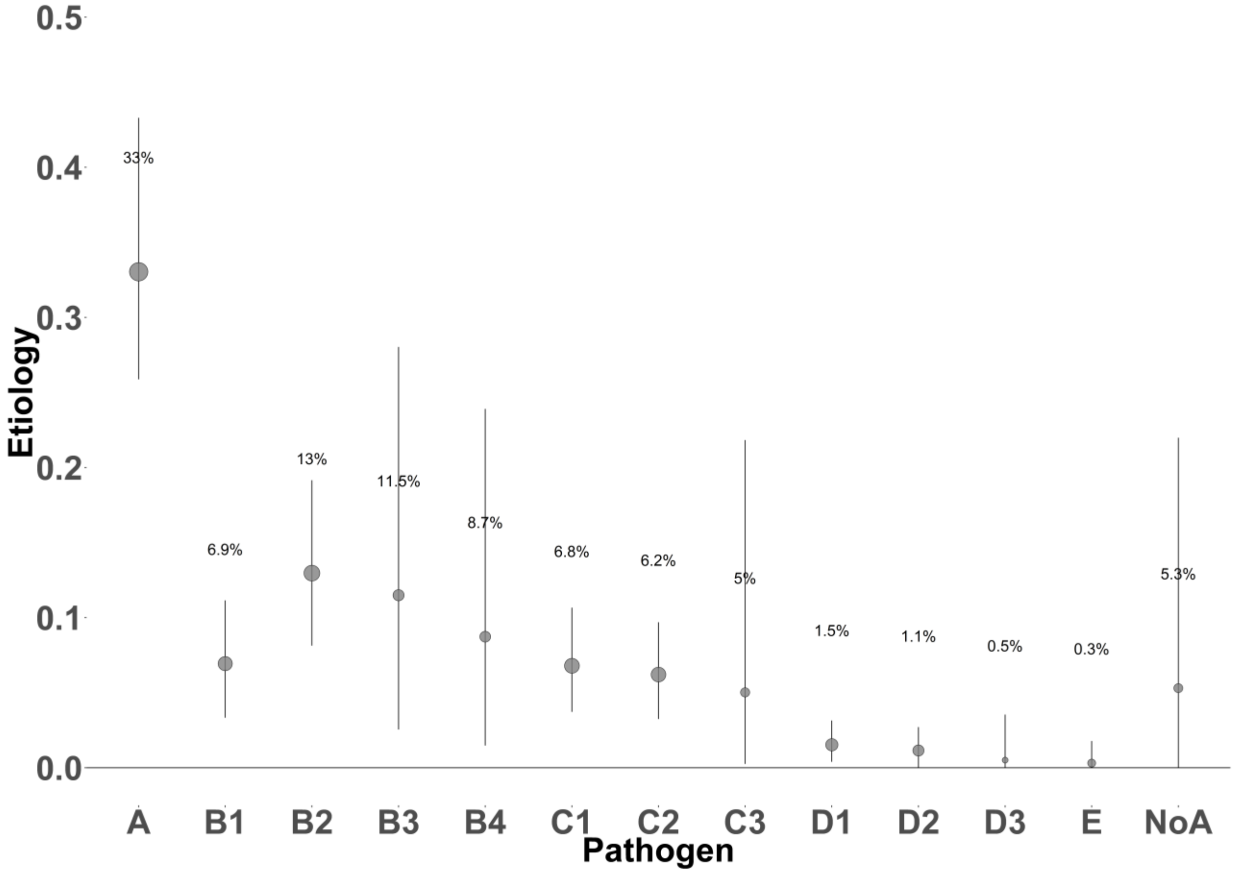

Supplemental Figure 5: Impact of poor specificity on etiologic estimation using PERCH Integrated Analysis (PIA) method: a sensitivity analysis.

PIA was performed on data (A) that had lower specificity (approximately 75%) for Bronze-standard measurements (e.g., NP/OP PCR). Results are shown in grey boxplots (B) and are compared to data with higher specificity (white boxplots) where specificity was >90% for all pathogens except B4, which by design had non-informative Bronze-standard data (also shown in Figure 4a). The boxplots show the distribution of results from PIA performed on 500 simulated datasets from each scenario. Each of the 500 datasets contained results from 600 cases and 600 controls and were created by random sampling from 'populations' with the case and control pathogen prevalences produced based on the true etiology proportions and sensitivity and specificity values of each scenario. The performance of PIA on the 500 simulated datasets with low specificity is shown in (C), and results from one randomly selected dataset are shown as a bubble plot in (D).

A. Characteristics of the data for the scenario where specificity of BrS measurements was lowered to 75%, thus changing ORs to 1.0-1.3

Pathogen	A	B1	B2	B3	B4	C1	C2	C3	D1	D2	D3	E	NoA
**True Etiology (%)**	36	10	10	10	10	5	5	5	1	1	1	0	6
**True BrS Sensitivity (%)**	75	75	75	/	75	75	75	/	75	75	/	75	/
**True BrS Specificity (%)**	75	75	75	/	27	75	75	/	75	75	/	75	/
**BrS positive, Cases (%)**	43	30	30	/	73.2	28	28	/	25.4	25.4	/	25.1	/
**BrS positive, Controls (%)**	25	25	25	/	73.0	25	25	/	25.1	25.1	/	24.9	/
**BrS Odds Ratio**	2.3	1.3	1.3	/	1.02	1.15	1.14	/	1.03	1.03	/	1.02	/
**True SS Sensitivity (%)**	/	15	/	15	15	15	/	15	15	/	15	/	/
**SS positive, Cases (%)**	/	1.5	/	1.5	1.5	0.75	/	0.74	0.14	/	0.15	/	/

Abbreviations: NoA, None of the above pathogens; BrS, Bronze-standard data (imperfect sensitivity and imperfect specificity, e.g., nasopharyngeal PCR); SS, Silver-standard data (imperfect sensitivity and perfect specificity, e.g., blood culture).

Pathogens A through D represent true pneumonia-causing pathogens that were tested for, Pathogen E represents a pathogen that was tested for but does not cause pneumonia, and NoA represents pathogens that cause pneumonia but were not tested for. Slashes indicate not applicable for the pathogen.

B. Distribution of etiologic estimates from PIA analyses of 500 simulated datasets for conditions when Bronze-standard specificity was high (>90%; white boxplots) versus low (75%; Grey)

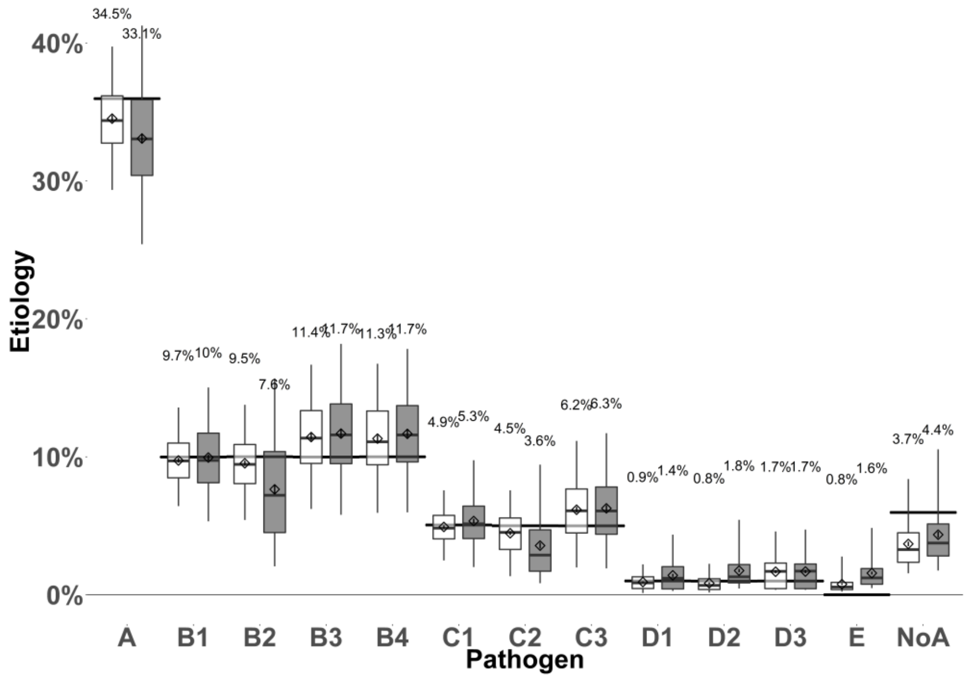

C. Performance of PIA when BrS specificity measurements are low (75%)

	Truth (%)	Estimate[a] (95% CI)	Bias[b]	Standard Deviation[c]	RMSE[d]	Coverage[e]	95% CI Length[f]
A	36	33.1 (22.9,45.9)	-2.9	4.2	5.1	99.2	23.0
B1	10	10.0 (4.6,16.8)	0.0	2.5	2.5	99.6	12.2
B2	10	7.6 (1.1,16.5)	-2.4	3.8	4.5	95.6	15.4
B3	10	11.7 (4.3,24.2)	1.7	3.1	3.5	100.0	19.9
B4	10	11.7 (4.3,23.9)	1.7	3.2	3.6	99.6	19.6
C1	5	5.3 (1.7,10.9)	0.3	1.9	1.9	99.4	9.2
C2	5	3.6 (0.1,10.8)	-1.4	2.5	2.8	96.0	10.7
C3	5	6.3 (1.6,15.6)	1.3	2.6	2.9	98.8	13.9
D1	1	1.4 (0.2,4.7)	0.4	1.2	1.2	98.2	4.5
D2	1	1.8 (0.0,7.1)	0.8	1.4	1.6	99.4	7.1
D3	1	1.7 (0.2,6.1)	0.7	1.3	1.5	98.8	6.0
E	0	1.6 (0.0,6.7)	1.6	1.2	2.0	0.0	6.7
NoA	6	4.4 (0.0,19.6)	-1.6	2.5	3.0	100.0	19.6

Abbreviations: CI, credible interval; RSME, root mean square error.

a. Estimate is defined as the mean etiologic estimate across the 500 datasets.

b. Bias is defined as the difference between the mean etiologic estimate and the true etiologic fraction values used to generate the data sets.

c. Standard deviation is defined as the square root of the variance across the 500 datasets.

d. Root mean square error (RMSE) is a measure of error, incorporating both bias and variance. RMSE is calculated as the average of the squared differences between the mean etiologic estimate and the truth for each of the 500 datasets.

e. Coverage is defined as the observed fraction of the 95% credible intervals that cover the true etiology fractions out of the 500 replications.

f. Average length of the 95% credible intervals across the 500 datasets.

D. Bubble plot of results from one randomly selected dataset from the scenario where Bronze-standard specificity was 75%.

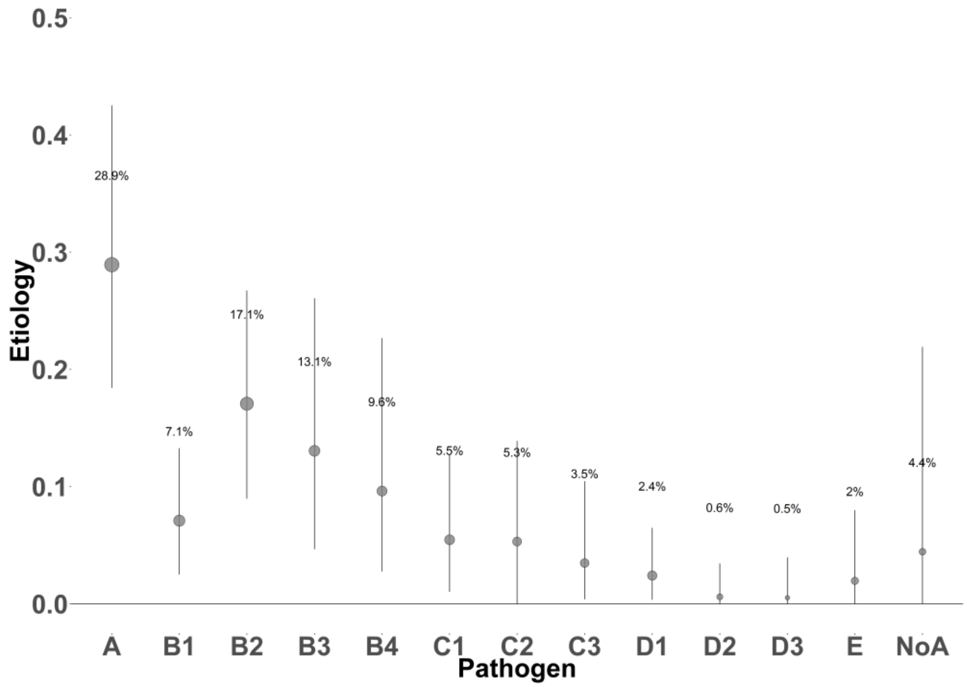

Supplement: Supplemental Tables and Figures [file cix144_suppl_Supplemental_Tables_and_Figures.pdf]
